# Hepatitis B Infection Awareness among Dental Graduate Students: A Cross Sectional Study

**DOI:** 10.1155/2014/389274

**Published:** 2014-10-29

**Authors:** Ramakrishnan Mahesh, Chandran Arthi, Samuel Victor, Seiramineni Ashokkumar

**Affiliations:** ^1^Department of Pediatric Dentistry, Saveetha Dental College & Hospitals, No. 162, Velapanchavadi Main Road, Poonamalle, Chennai 600077, India; ^2^Department of Periodontics, Meenakshi Ammal Dental College & Hospitals, No. 162, Velapanchavadi Main Road, Poonamalle, Chennai 600077, India

## Abstract

Hepatitis B virus transmission in a dental setting more commonly occurs due to inadequate/improper use of safety measures by the dentist. This particular study evaluated the hepatitis B virus infection related awareness among dental graduate students in a University Dental College, India. A validated questionnaire regarding the awareness about hepatitis infection and various infection control measures was distributed among the students of different year of study in undergraduate bachelor dental graduate program. The data extracted were tabulated and analyzed. Final year students showed an increased awareness when compared to third year students. There is need for improving the knowledge among the nonclinical students, mainly on transmission of virus through salivary contact. The overall awareness among the students is only fairly satisfying, which signifies the need for continued infection control education among the students.

## 1. Introduction

Hepatitis B virus infection (HBV) is an inflammatory disease of liver due to double stranded virus of the hepadnaviridae family [1]. Hepatitis B infection possesses a major health concern and is the most common blood borne viral infection, placing health care workers and medical and dental professionals at higher occupational risk [1]. The other mostly common communicable diseases include human immunodeficiency virus (HIV) and hepatitis C virus (HCV).

The possible forms of transmission of hepatitis virus include unprotected sexual contact, blood transfusion, reuse of contaminated needles, and vertical transmission from mother to child during pregnancy [2]. In dental setting the most common mode of transmission is from percutaneous exposure (needle stick injuries) and also from contact with blood or saliva of infected patients. The possibility of HBV transmission from exposure to saliva and gingival crevicular fluid has been confirmed, which makes the oral health care professionals more vulnerable for hepatitis infection [3].

Nearly two billion people in the world have been infected by HBV and there are nearly 350 million people who are chronic carriers [4]. HBV infections are 50 to 100 times more infectious than HIV [5]. Hepatitis B and hepatitis C infection can become persistent and show the way to cirrhosis of liver and even liver cancer. It is mainly acquired in the course of contaminated needles or tainted blood products and infection patterns are diffuse [6]. Among the professionals, dentists are placed in high risk group as actual sufferers and carriers with a grim picture. It is of prime importance for all dental schools, medical staff, and dental staff to conduct talks and create awareness about hepatitis B infection.

India has the intermediate endemicity of hepatitis B virus with surface antigen (HBsAg) prevalence between 2% and 10% among the population studied [7]. In India about four percent of the population was estimated to be HBV carriers giving a total pool of approximately 36 million carriers [8].

Chronic infection with hepatitis B may be either asymptomatic or may be associated with a chronic inflammation of the liver (chronic hepatitis), leading to a cirrhosis over a period of several years [8]. Studies have shown that the risk of exposure for general dentists is about three to four times greater and for nonimmunized surgical specialists about six times greater than that of the general population [9]. In the dental setting, there are special circumstances and opportunities which can lead to the transmission of such organisms to dental healthcare professionals and to dental clinical students [10].

The incidence of HBV can be reduced by giving proper education regarding its transmission and immunizations to the public, all healthcare workers (HCV), and students [11]. There are no adequate data on the awareness of hepatitis among dental college students in India. Hence this forms the base of the present study which aimed to analyze the awareness of hepatitis B infection among the clinical students in a private university.

## 2. Materials and Methods

### 2.1. Approval

This is an institutional based study conducted to determine the awareness on hepatitis B infection among dental students in University Dental College. The study protocol was reviewed by the institutional ethical board.

### 2.2. Year of Study

The study was conducted during the academic year in July 2013.

### 2.3. Study Population and Location

This study is conducted among the dental students who are attending the third year, Final year, and internship (fifth year trainee) of graduate program in Saveetha Dental College and Hospital.

### 2.4. Inclusion and Exclusion Criteria

The students who were present at the day of the particular study and were willing to participate were included in the study and those who were not willing to participate were not included in the study.

### 2.5. Study Sample Size

All the third year, final year, and intern students who were present at the day of study were considered as the sample size for the study.

### 2.6. Questionnaire

A validated questionnaire [12] was distributed among all the students of the study. This is a self-reported questionnaire which takes about 10 mins for completion. All the questions were given two options (yes/no). This included questions about the awareness on hepatitis B infection and the preventive measures taken by the dentist to protect both patients and dentists from hepatitis infection.

### 2.7. Statistical Analysis

Both the questionnaire and oral examination forms were manually checked for completion of data. All data were entered in a data entry form which was transferred to SPSS software (SPSS, version 17 for Windows). Analysis of variance (ANOVA) was performed for each variable to assess whether significant differences were observed between the three years of study and also within groups. *P* < 0.05 was considered to be statistically significant.

## 3. Results

A total of 150 students participated in the study. Of these 34 (22.7%) students were male and 116 (77.3%) were female. The students were equally distributed among all the three-year groups.

Table 1 shows the distribution of answers to each question. The question regarding possible transmission of HBV through contact with saliva, only 23 (25.3%) of the third year students were aware of the fact while 45 (30%) of the final year students and 41 (27.3%) of the interns were already aware of salivary transmission which showed a significant difference (*P* = 0.000). In response to the question regarding confirmed risk of HBV transmission through dental treatments 30 (20%) of the third year students, 35 (23.3%) of the final year students, and 32 (21.3%) of the interns were aware of the possible risk during the procedure. Regarding high risk of HBV infection for dentist 24 (16%) of the third year students, 40 (26.7%) of the final year students, and 41 (27.3%) of the interns were aware of the high risk, which showed statistically significant difference between various academic years (*P* < 0.05). In response to the question on needle stick injuries 30 (20%) of the third year students, 34 (22.75%) of the final year students, and 35 (15%) of the interns reported having such unintentional procedures. This variable did not show a statistically significant difference between various academic years (*P* = 0.541).

 Questions regarding high risk of transmission of HBV than HIV through needle stick injury 26 (17.3%) of the third year students, 31 (20.7%) of the final year students, 25 (16.7%) of the interns replied that risk for HIV transmission is more. Twenty three 23 (15.3%) of the third year students, 27 (18%) of the final year students, and 36 (24%) of the interns were aware that HBV transmission from dentist to patient can be prevented with the use of gloves which did not show a statistically significant difference between academic years (*P* = 0.026).

 To question regarding the level of knowledge about universal precaution 6 (4%) of the third year students, 5 (3.3%) of the final year students, and 6 (4%) of the interns were not aware about the precautions. Five (3.3%) of the third year students, 14 (9.3%) of the final year students, and 19 (12.7%) of the internship students have heard something about universal precautions. Nineteen (12.7%) of the third year students, 27 (18%) of the final year students, and 21 (14%) of the interns were being educated about universal precaution. Twenty (13.3%) of the third year students, 4 (2.7%) of the final year students, and 4 (2.7%) of the interns were following an executable protocol for universal precaution. This variable shows a significant difference between the academic years (*P* = 0.002).

Table 2 shows the comparison between and within groups on the response to each question. The question regarding salivary transmission of HBV showed a significant difference among the students of various years. The question regarding higher predominance of dentist among the population for HBV infection also showed a significant difference (*P* < 0.05). Regarding the universal precaution there was a significant difference between the various year students (*P* < 0.05).

## 4. Discussion

The overall level of knowledge about viral hepatitis among the various years is fairly satisfactory. The level of knowledge regarding HBV was fairly good among final and the interns when compared to 3rd year students. There is no formal school based health education for students in India, and this may also be considered as one of the important reasons for lower knowledge of hepatitis B among 3rd year students. The dental curriculum in India is such that students enter the clinical practice only in third year of study. The first two years they are trained in the basic medical sciences. It is because of this the study population included students only in the clinical year of study. To our knowledge, this is the first study investigating this topic among dental students in Chennai, India.

Based on the results of this study, we can infer that there is need for improving the knowledge of HBV infection among the nonclinical students. When a student enters the final year they are exposed more to the clinical situations and complications and hence exhibit improved knowledge when compared to third year students. A significant difference was seen among the third year students when asked regarding the transmission of HBV through salivary contact. Most of the third year students were not aware of the increased risk among dental professionals when compared to general population. Since the students enter the clinical in the third year of study, proper immunization and education among them can minimize the early transmission of HBV among the dental students. The level of knowledge and compliance with infection control measures was poor among the students. Attributable reasons could be inadequate training for infection control measures, inadequate supply of personal protective equipment, and carelessness [13].

Proper hand washing and use of barriers such as gloves, gowns, and mask are the main components of standard precautions which can minimize mucocutaneous exposures. Reducing the manipulation of manual sharp instruments can also prevent occupational injuries. The use of puncture-resistant containers for sharp disposal is also an effective strategy. Use of protective eye wares and face mask can help in preventing blood or saliva contact during the procedure. Indirect transmission of hepatitis B virus can also occur through the dental instruments hence a proper method of sterilization needs to be educated among clinical students. Vaccination against hepatitis B is recommended for all the dental students before they start their clinical phase and for susceptible dentists and dental auxiliary staff [14, 15]. It is recommended that a policy be implemented for complete vaccination and health education for all dental students in first year in all dental colleges. However, antibody titers should be routinely checked among all vaccinated students because in some cases there are chances of nonresponse to the first series of vaccination. Work practice management and reinforcement of universal precautions are to be done periodically. It is of prime importance for all dental schools to conduct talks and create awareness about hepatitis B infection. The study highlights the need for implementing separate course in dental curriculum on communicable diseases in the first year of dental school. At the end of our study the students were given a lecture and demonstration on various universal precaution methods which they should follow when treating any patients. The students who were not vaccinated were referred for immediate vaccination.

Our research should be viewed with the following limitations in mind. Firstly, all collected data were self-reported and therefore not verifiable. In particular, there was some inconsistency in the collected data (only yes or no option was given for all questions), indicating that some of the reported information was unreliable. Since it is a cross-sectional study the knowledge at that point of study was only considered. Lastly, because of the nonrandom sampling, our conclusions have limited generalizability to other dental college students in India. However the results of the study can be used as a baseline for enhancing the knowledge about infection control by conducting continuing dental education program.

Although dentists and dental students can be exposed to the human immunodeficiency virus (HIV), hepatitis B virus (HBV), and hepatitis C virus (HCV) in their work environment, this particular study evaluated the awareness only on hepatitis B virus since it is most commonly underdiagnosed and the risk of transmission is much higher compared to other communicable diseases.

## 5. Conclusion

Although the overall level of knowledge about viral hepatitis among the various years is fairly satisfactory, the students entering the third year of clinical study have less knowledge of HBV infection. This finding highlights the necessity of continuous infection control education.

## Figures and Tables

**Table 1:** Showing the distribution of answers to each question.

Questions	Third year	Final year	Interns	Total	*P* value
	*N* (%)	*N* (%)	*N* (%)	*N* (%)	
Q1. HBV transmitted through saliva					
Yes	23 (25.3%)	45 (30%)	41 (27.3%)	109 (72.2%)	**P** < 0.05
No	27 (18%)	5 (3%)	9 (6%)	41 (27.3%)	
Q2. HBV from dentist to patient					
Yes	27 (18%)	27 (18%)	38 (25.3%)	92 (61.3%)	
No	23 (15.3%)	23 (15.3%)	12 (8%)	58 (38.7%)	0.033
Q3. HBV from patient to patient.					
Yes	32 (21.3%)	40 (26.7%)	42 (28%)	114 (76%)	
No	18 (12%)	10 (6.7%)	8 (5.3%)	18 (12%)	0.046
Q4. Risk of HBV through dental treatments					
Yes	30 (20%)	35 (23.3%)	32 (21.3%)	97 (64.7%)	
No	20 (13.3%)	15 (10%)	18 (12%)	53 (35.3%)	0.580
Q5. Dentists are at higher risk of HBV infection than general population					
Yes	24 (16%)	40 (26.7%)	41 (27.3%)	105 (70%)	
No	26 (17.3%)	10 (6.7%)	9 (6%)	45 (30%)	**P** < 0.05^*^
Q6. Considerable amounts of dentists experience needle stick injury frequently					
Yes	30 (20%)	34 (22.7%)	35 (15%)	99 (66%)	
No	20 (13.3%)	16 (10.7%)	15 (10%)	51 (34%)	0.541
Q7. High risk of HBV than HIV transmission through needle stick injury					
Yes	26 (17.3%)	31 (20.7%)	25 (16.7%)	82 (54.7%)	
No	24 (16%)	19 (12.7%)	25 (16.7%)	68 (45.3%)	0.440
Q8. High risk of HBV than HCV transmission through needle stick injury					
Yes	27 (18%)	30 (20%)	32 (23.3%)	89 (59.3%)	
No	23 (15.3%)	20 (13.3%)	18 (12%)	23 (15.3%)	0.597
Q9. HBV transmission from patient to patient can be prevented with the use of gloves					
Yes	27 (18%)	30 (20%)	31 (20.7%)	88 (58.7%)	0.704
No	23 (15.3%)	20 (13.3%)	19 (12.7%)	62 (41.3%)	
Q10. HBV transmission from dentist to patient can be prevented with the use of gloves					
Yes	23 (15.3%)	27 (18%)	36 (24%)	86 (57.3%)	
No	27 (18%)	23 (15.3%)	14 (9.3%)	64 (42.7%)	0.026
Q11. Knowledge about universal precautions					
Do not know	6 (4%)	5 (3.3%)	6 (4%)	17 (11.3%)	
Heard something	5 (3.3%)	14 (9.3%)	19 (12.7%)	38 (25.3%)	**P** < 0.05^*^
Being educated	19 (12.7%)	27 (18%)	21 (14%)	67 (44.7%)	
Following executable protocol	20 (13.3%)	4 (2.7%)	4 (2.7%)	28 (18.7%)	

^*^Values which show significant result (*P* < 0.05).

**Table 2:** Comparison between and within groups on the response to each questions using ANOVA.

Variable	Sum of squares	df	Mean square	*F*	Sig
Q1					
Between groups	4.493	2	2.74	16.62	**P** < 0.05^*^
Within Groups	24.3	147	0.16		
Total	29.79	149			
Q2					
Between groups	1.61	2	0.80	3.49	0.033
Within Groups	33.96	147	0.23		
Total	35.57	149			
Q3					
Between groups	1.12	2	0.56	3.14	0.046
Within Groups	26.24	147	0.18		
Total	27.36	149			
Q4					
Between groups	0.25	2	0.13	0.55	0.580
Within Groups	34.02	147	0.23		
Total	34.27	149			
Q5					
Between groups	3.64	2	1.82	9.60	**P** < 0.05^*^
Within Groups	27.86	147	0.19		
Total	31.5	149			
Q6					
Between groups	0.28	2	0.14	0.62	0.541
Within Groups	33.38	147	0.22		
Total	33.66	149			
Q7					
Between groups	0.41	2	0.20	0.82	0.440
Within Groups	36.76	147	0.25		
Total	37.17	149			
Q8					
Between groups	0.25	2	0.12	0.51	0.597
Within Groups	35.94	147	0.24		
Total	36.19	149			
Q9					
Between groups	0.17	2	0.08	0.35	0.704
Within Groups	36.20	147	0.24		
Total	36.37	149			
Q10					
Between groups	1.77	2	0.88	3.73	0.026
Within Groups	34.92	147	0.23		
Total	36.69	149			
Q11					
Between groups	9.85	2	4.92	6.51	**P** < 0.05^*^
Within Groups	111.24	147	0.75		
Total	121.09	149			

^*^Values which show significant result (*P* < 0.05).

One way ANOVA test.
